# Dietary data of a highly biodiverse anuran assemblage in the Ecuadorian Amazon

**DOI:** 10.1016/j.dib.2022.108720

**Published:** 2022-11-09

**Authors:** Pablo A. Menéndez-Guerrero, Santiago R. Ron, Sofía Carvajal-Endara

**Affiliations:** a Museo de Zoología (QCAZ), Escuela de Biología Facultad de Ciencias Exactas y Naturales, Pontificia Universidad Católica del Ecuador, Quito, Ecuador; b Centro de Investigación en Biodiversidad y Cambio Climático (BioCamb), Ingeniería en Biodiversidad y Recursos Genéticos, Facultad de Ciencias del Medio Ambiente, Universidad Tecnológica Indoamérica, Av. Machala y Sabanilla, Quito, Ecuador

**Keywords:** Amphibia, Biotic interactions, Diet composition, Gastrointestinal contents, Interaction network, Prey items, Trophic niche, Yasuní National Park

## Abstract

This dataset reports the diet composition of a highly diverse anuran assemblage in the Ecuadorian Amazon region. In 2001 we examined the diet of an assemblage of frogs from Yasuní National Park. We describe the diet of 396 adult individuals, belonging to 35 species, based on their gastrointestinal contents. Using a stereoscopic microscope, we were able to identify 4085 prey items, and classified them in 71 categories. Also, we used a digital caliper to measure the size and estimate the volume of prey items that were found intact. In addition to diet composition, we provide information of all specimens that were examined including, museum number, family name, species name, and place and date of collection. Finally, we present an anuran–prey interaction network figure to visualize species interactions. This is the first report of the diet composition of an anuran assemblage from Yasuní National Park. It contributes to the understanding of trophic ecology of frog assemblages and the functional role of frogs in Amazonian ecosystems. In addition, our dataset helps to fill the great knowledge gap that exists about ecological interactions in the tropics.

**Specifications Table**

**Table:** 

Subject	Biological Sciences
Specific subject area	Systematics, Ecology and Behavior
Type of data	Tables Figure
How the data were acquired	We report the gastrointestinal content composition of 35 anuran species collected at Yasuní National Park in the Ecuadorian Amazon Region between 1992 and 2001. We examined all anurans available in the Museo de Zoología (QCAZ) of Pontificia Universidad Católica del Ecuador in Quito. We dissected 396 adult specimens to extract and describe their gastrointestinal contents. We identified prey items to the lowest taxonomic level possible using a stereoscopic microscope. We also measured the length and width of intact prey items using a digital caliper to estimate their volume. In addition, we built a bipartite network to visualize anuran–prey interactions.
Data format	Raw Filtered
Description of data collection	We examined adult anurans that were collected at Yasuní National Park between 1992 and 2001. The number of specimens examined per species varied from 1 to 36, according to their availability in the museum collection. We excluded data from anuran specimens for which taxonomic identification to species level was unfeasible.
Data source location	Location: Yasuní National Park Region: Amazonian Region Country: Ecuador Landmark: Pontífica Universidad Católica del Ecuador's scientific research station (PUCE-YSRS) (-0.674792°; -76.396897°)
Data accessibility	Repository name: Dryad Data identification number: doi:10.5061/dryad.wdbrv15rx Direct URL to data: https://datadryad.org/stash/dataset/doi:10.5061/dryad.wdbrv15rx

## Value of the Data

• Our dataset is the first report of anurans trophic interactions at Yasuní National Park, one of the most species-rich anuran assemblages worldwide.
• Our dataset could be used to better understand anuran trophic ecology, as well as the functional roles of anurans in tropical ecosystems.
• Our dataset could be used to evaluate anuran species niche breadth and their vulnerability to extinction.
• Researchers interested in ecological interactions and conservation of tropical ecosystems can benefit from these data. It can support future studies on the loss of ecological interactions and ecosystem services due to habitat deterioration or climate change.

## 1 Objective

We generated this data to study the trophic ecology of anurans from Yasuní National Park [1]. Yasuní National Park, located in the Ecuadorian Amazon Region, is among the most biodiverse places on Earth [2] and constitutes the largest protected region in continental Ecuador with an approximate surface area of 9,820 Km^2^ [3]. This area holds a remarkably high anuran diversity, so far 135 species have been reported [4], yet the ecology of most of these species remains unknown.

## 2 Data Description

Our dataset consists of four tables, shared in a public repository (https://datadryad.org/stash/dataset/doi:10.5061/dryad.wdbrv15rx) and one figure presented here that describe the diet composition of an anuran assemblage from Yasuní National Park. Table 1 is a matrix summarizing anurans-prey interactions, it shows the number of each prey category found within the gastrointestinal contents of anuran species collected from 1992 to 2001 at Yasuní that were available in the Museo de Zoología (QCAZ) of the Pontificia Universidad Católica del Ecuador. In columns, names correspond to 71 prey categories in alphabetic order. In rows, names correspond to 35 anuran species in alphabetic order and grouped by family. Anuran species names are abbreviated as follow (Ama = *Amazophrynella*, All = *Allobates*, Ame = *Ameerega*, Hyl = *Hyloxalus*, Aga = *Agalychnis,* Boa = *Boana*, Den = *Dendropsophus*, Nyc = *Nyctimantis*, Ost = *Osteocephalus*, Phy = *Phyllomedusa*, Sci = *Scinax*, Tra = *Trachycephalus*, Eda = *Edalorhina*, Eng = *Engystomops*, Pri = *Pristimantis*).

Table 2 is a matrix showing the volume in mm^3^ of each prey category. Since volume was estimated only from intact prey items, there are prey categories found in the gastrointestinal contents that lack volume data. Column names correspond to prey categories and row names to anuran species as in Table 1.

Table 3 is a matrix showing the number of intact items per prey category which were used to estimate the volume presented in Table 2. Column names correspond to prey categories and row names to anuran species as in table 1. In Table 4, we summarized information of all specimens that were examined to build this dataset. We included here the museum number, family name, species name, date and place of collection and collectors. All anuran specimens and their gastrointestinal contents are available at the Museo de Zoología (QCAZ) of the Pontificia Universidad Católica del Ecuador.

**Fig. 1:**
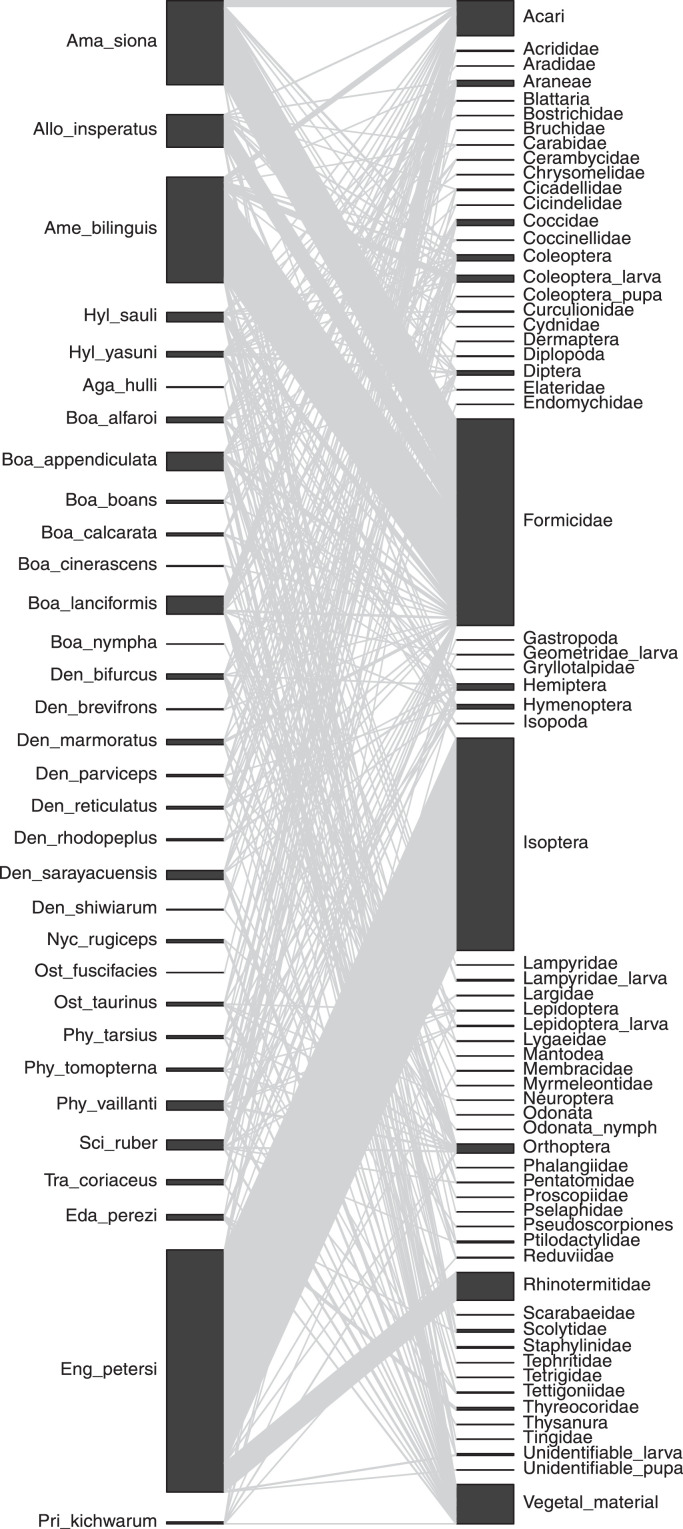
Bipartite anuran-prey interaction network of an anuran assemblage from Yasuní National Park. The numbers of prey items found within the gastrointestinal contents are shown as links (grey lines) connecting black bars at the left (anuran species) and the right (prey items). Anuran species names are abbreviated as follow (Ama = *Amazophrynella*, All = *Allobates*, Ame = *Ameerega*, Hyl = *Hyloxalus*, Aga = *Agalychnis*, Boa = *Boana*, Den = *Dendropsophus*, Nyc = *Nyctimantis*, Ost = *Osteocephalus*, Phy = *Phyllomedusa*, Sci = *Scinax*, Tra = *Trachycephalus*, Eda = *Edalorhina*, Eng = *Engystomops*, Pri = *Pristimantis*). The bar length represents the number of interactions observed per species and prey categories. Anuran species and prey item categories are shown in the same order as presented in Table 1.

Finally, in Fig. 1 we present an anuran–prey interaction network, where the numbers of each prey category found per species are shown as links anuran species and prey categories. This figure was built using data provided in Table 1.

## 3 Experimental Design, Materials and Methods

To describe the diet composition of anurans from Yasuní we used specimens that were collected during several independent biotic surveys performed between 1992 and 2001 that were available at the Museo de Zoología (QCAZ) of Pontificia Universidad Católica del Ecuador in Quito. Most collections were made between 1994 and 1995. We examined 35 species including six families: Aromobatidae, Bufonidae, Dendrobatidae, Hylidae, Leptodactylidae, and Strabomantidae. The number of individuals used to report the diet composition of each species varied from 1 to 36 according to their availability at the QCAZ collection at the time of the analysis (October 2000 - May 2001). We updated taxonomic identification of anurans using specimen information available at the Anfibios del Ecuador BIOWEB portal (https://bioweb.bio/faunaweb/amphibiaweb/) [4] following taxonomy from AmphibiaWeb (https://amphibiaweb.org/) [5]. We excluded from this report 35 specimens for which taxonomic identification at species level is uncertain. Most specimens were collected between 1994 and 1995, by hand, during day and night. Specimens were not captured with the objective of performing a dietary description, and thus the time between collection and preservation varied. During this time, digestion of preys continue within gastrointestinal tract of anurans, which could affect the quantity and quality of prey items recovered [6]. However, a standard practice among these surveys was to euthanize and preserve animals as soon as possible after capture. Anurans were euthanized using lidocaine, fixed in 10% formalin between 5-12 hours after collection, and preserved in ethanol 70-75%.

We dissected 396 specimens to extract their digestive tract. After splitting the stomach from the intestine section, we stored them apart in ethanol 70%. All dissected specimens and their gastrointestinal contents are available in individual vials at the QCAZ museum. We examined the gastrointestinal content under a Nikon stereomicroscope (SMZ-800) and identified 4085 prey items that were classified under 71 categories. Insects were identified to the lowest taxonomic category possible, mainly families and orders, following Borror et al. [7] taxonomic classification. We also identified insect larvae, pupae, and nymphs to Family or Order when possible and classified them under separate categories (e.g. Geometridae larva), otherwise they were classified under broad categories such as “Unidentifiable larva”. We categorized flowers and seeds as “Vegetal material” but excluded leaves. When prey items were found intact or nearly intact (i.e. when available fragments of a prey item allowed us to determine its general shape), we measured their length in mm (excluding antennae, jaws, and ovipositors) and width in mm (at the midpoint and excluding legs) using a digital caliper (Fowler & NSK; precision 0.01mm). These measurements we applied to the prolate spheroid formula [8,9] to estimate prey volume:

$$V=4/3\pi \left(\text{length}/2\right)*{\left(\text{width}/2\right)}^{2}$$

Finally, to visualize anuran-prey interactions we built a bipartite interaction network based on the number of each prey items found per anuran species, using the “bipartite” R package v. 2.17 [10] in the R environment [11]. To plot the network we used the function *plotweb (),* excluding anuran species lacking identifiable prey items (*Osteocephalus mutabor, Scinax cruentommus* and *Scinax garbei*) and leaving the order of the network elements as given by the matrix provided in Table 1.

## Ethics Statements

Our data was obtained from museum specimens therefore an ethic approval for surveys was not required.

## CRediT authorship contribution statement

**Pablo A. Menéndez-Guerrero:** Conceptualization, Methodology, Data curation, Writing – review & editing. **Santiago R. Ron:** Conceptualization, Data curation, Writing – review & editing. **Sofía Carvajal-Endara:** Writing – review & editing.

## Declaration of Competing Interest

The authors declare that they have no known competing financial interests or personal relationships that could have appeared to influence the work reported in this paper.

The authors declare the following financial interests/personal relationships which may be considered as potential competing interests.

## Data Availability

Dietary data of an anuran assemblage from Yasuní National Park (Original data) (Dryad).
